# The complete mitogenome and phylogenetic analysis of Indian driftfish, *Cubiceps squamiceps* (Scombriformes: Nomeidae)

**DOI:** 10.1080/23802359.2021.1914235

**Published:** 2021-05-10

**Authors:** Ying Peng, Jiasheng Li, Kun Zhang, Yifan Liu, Hongqiang Wang, Xuepeng Li, Hua Zhang, Bingjian Liu, Kuo Tian

**Affiliations:** a Marine Science and Technology College, Zhejiang Ocean University, Zhoushan, China; b Zhoushan Hospital of Zhejiang Province, Zhoushan, China; c School of Ocean, Yantai University, Yantai, China; d Key Laboratory of Tropical Marine Bio-resources and Ecology, Chinese Academy of Sciences, Beijing, China; e School of Fishery, Zhejiang Ocean University, Zhoushan, China

**Keywords:** Complete mitogenome, *Cubiceps squamiceps*, phylogeny

## Abstract

The Indian driftfish (*Cubiceps squamiceps*) is one of the most important commercial fish species in China, Japan and India. The complete mitogenome of *Cubiceps squamiceps* was determined in this study. The assembled mitogenome was 16,507 bp and consisted of 13 protein-coding genes, 22 tRNAs, two rRNAs, and a control region. Nucleotide composition of the complete mitogenome was 27.5% A, 28.5% C, 17.5% G, and 26.5% T, with an A + T bias of 53.9%. The maximum-likelihood tree based on 13 protein-coding genes showed that *Cubiceps pauciradiatus* and *Psenes pellucidus* were the closest to *C. squamiceps*.

*Cubiceps squamiceps* belongs to genus *Cubiceps*, family Nomeidae, order Stromateoidei. It is widely distributed in the coast of India-Western Pacific (Zhao et al. 2016). Previous studies on *C. squamiceps* were mainly related to its distribution and biological characteristics. For example, Kim et al. (1988) found five new fish species collected from the southern coastal waters of Korea, one of which was *C. squamiceps*. Guang et al. (2017) determined the concentrations of heavy metals (Cd, Pb, Cr, Ni, Cu and Zn) in *C. squamiceps* and found that concentrations of heavy metals in *C. squamiceps* were below their acceptable daily upper limit, suggesting human consumption of these wild fish species may be safe. The complete mitochondrial genome was considered as a useful tool for species identification, phylogeny and evolution (Zhang and Georges 2014). There were a few researches on evolution and phylogenetic analysis of this fish. In this study, we described the complete mitochondrial genome of *C. squamiceps* and explored the phylogenetic relationship within Scombriformes, which can provide an important dataset for a better understanding of the mitogenomic diversities.

Firstly, samples of *C. squamiceps* were collected from the East China Sea (N 27°49′52.12″, E 121°10′31.21″) and stored in a laboratory of Zhejiang Ocean University with accession number 20200513bj19. The email of the people in charge of the collection is 1301909511@qq.com. Total genomic DNA was extracted using standard phenol-chloroform extraction procedure (Sambrook and Russell 2006). Subsequently, based on the existing complete mitochondrial gene of *Psenes pellucidus* (NC_021619), 19 pairs of primers were designed. The samples were amplified by PCR and sequenced using Sanger sequencing technology. The complete mitochondrial genome was annotated using Sequin version 15.10 (http://www.ncbi.nlm.nih.gov/Sequin) and tRNAscan-SE version 2.0 (http://trna.ucsc.edu/tRNAscan-SE/; Lowe and Eddy 1997). The mitochondrial genome of *C. squamiceps* has been stored in the GenBank with accession number MW401268. The complete mitogenome of the *C. squamiceps* was 16,507 bp in length including two rRNA genes, 13 protein-coding genes, 22 tRNA genes and a control region, which was similar to other typical vertebrate mitochondria (Boore 1999). Compared with the sequence stored in GenBank with Acc Nos. KT_361215 and NC_029845, we had provided a new sequence with a difference of 3 bases. The different sequence numbers of the same species may be caused by the different sampling sites. The overall base composition was 27.5% (A), 26.5% (T), 28.5% (C), and 17.5% (G) with a slight A + T bias (53.9%), which was similar to other typical Nomeidae fish mitogenomes (Wei et al. 2014). 12 genes started with ATG while only COI started with GTG among the 13 protein-coding genes. Seven genes shared the termination codon TAA including ND1, COI, ATP8, ATP6, COIII, ND4L and ND5, and the gene with TAG as the stop codon was ND6. The remaining protein-coding genes with incomplete termination codons were COII (T–), ND2 (TA-), ND3 (T–), ND4 (T–), and Cytb (T–). Besides, nine genes were coded on the L-strand including tRNA-Gln, tRNA-Ala, tRNA-Asn, tRNA-Cys, tRNA-Tyr, tRNA-Ser, tRNA-Glu, tRNA-Pro and the ND6 gene, the others were encoded on the H-strand which was consistent with other fish mitochondrial genes (Yukai et al. 2019).

Maximum-Likelihood phylogeny was constructed based on 13 protein-coding genes of *C. squamiceps* and other 15 Scombriformes using the software PhyML 3.0 (Guindon et al. 2010). The most suitable nucleotide sequence model GTR + I + G was selected through MrModeltest 2.3 based on the Akaike Information Criteria (AIC) (Yamaoka et al. 1978). The phylogenetic analysis showed that *Cubiceps pauciradiatus* and *Psenes pellucidus* were the closest to *C. squamiceps* which was consistent with the result of Zhao et al. (2016). The complete mitochondrial genome sequence of the *C. squamiceps* can provide an important dataset for a better understanding of the mitogenomic diversities, evolution in *C. squamiceps* and facilitating the identification of the species (Figure 1).

**Figure 1.**
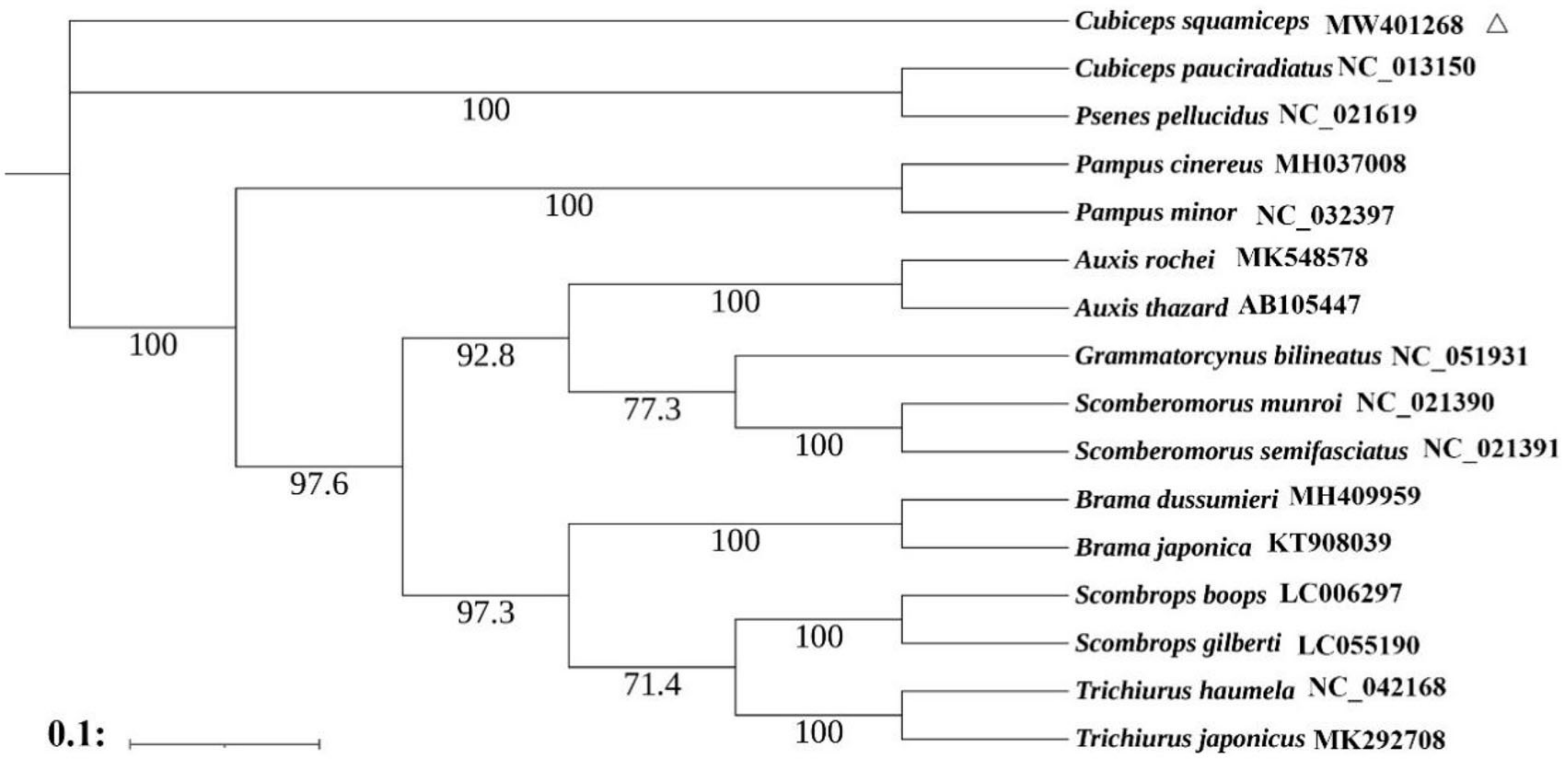
Maximum-likelihood phylogenetic tree of 16 Scombriformes fishes based on 13 protein-coding genes. Bootstrap support values are shown on the nodes and numbers following scientific names are GenBank accessions.

## Data Availability

Mitochondrial genome sequence can be accessed via accession number MW401268 (https://www.ncbi.nlm.nih.gov/nuccore/MW401268) in the NCBI GenBank.

## References

[CIT0001] Boore JL. 1999. Animal mitochondrial genomes. Nucleic Acids Res. 27(8):1767–1780. 10101183 10.1093/nar/27.8.1767 PMC148383

[CIT0002] Guang GY, Lin Q, Hui HH, Gen WL, Jia NJ, Yan DF. 2017. Heavy metals in fish tissues/stomach contents in four marine wild commercially valuable fish species from the western continental shelf of South China Sea. Mar Pollut Bull. 114(2):1125–1129. 27765407 10.1016/j.marpolbul.2016.10.040

[CIT0003] Guindon S, Dufayard J-F, Lefort V, Anisimova M, Hordijk W, Gascuel O. 2010. New algorithms and methods to estimate maximum-likelihood phylogenies: assessing the performance of PhyML 3.0. Syst Biol. 59(3):307–321. 20525638 10.1093/sysbio/syq010

[CIT0004] Kim KH, Kim YU, Kim YS. 1988. Five species of fish new to Korean Waters. Korean J Fish Aquat Sci. 21:105–112.

[CIT0005] Lowe T, Eddy S. 1997. tRNAscan-SE: a program for improved detection of transfer RNA genes in genomic sequence. Nucleic Acids Res. 25(5):955–964. 9023104 10.1093/nar/25.5.955 PMC146525

[CIT0006] Sambrook J, Russell DW. 2006. Purification of nucleic acids by extraction with phenol:chloroform. Csh Protoc. 2006(1):pdb.prot4455. 22485786 10.1101/pdb.prot4455

[CIT0007] Wei T, Zhang B, Sun Y. 2014. Structure comparison of control region in Stromateoidei and complete mitochondrial genome of the bluefin driftfish *Psenes pellucidus* (Perciformes, Nomeidae). Mitochondrial DNA. 25(5):355–356. 23815326 10.3109/19401736.2013.803091

[CIT0008] Yamaoka K, Nakagawa T, Uno T. 1978. Application of Akaike’s information criterion (AIC) in the evaluation of linear pharmacokinetic equations. J Pharmacokinet Biopharm. 6(2):165–175. 671222 10.1007/BF01117450

[CIT0009] Yukai Y, Xiaolin H, Heizhao L, Tao L, Wei Y, Zhong H. 2019. The complete mitochondrial genome of *Chaetodon wiebeli* (Chaetodontiformes, Chaetodontidae). Mitochondrial DNA B Resour. 4(2):3145–3146. 33365891 10.1080/23802359.2019.1667894 PMC7706881

[CIT0010] Zhang X, Georges A. 2014. A complete mitochondrial genome sequence for the Australian turtle, *Chelodina longicollis*, obtained using 454-pyrosequencing. Conserv Genet Resour. 6:555–557.

[CIT0011] Zhao M, Ma H, Ma C, Zhang H, Zhang X, Meng Y, Wei H, Chen F, Ma L. 2016. The complete mitochondrial genome and gene organization of *Cubiceps squamiceps* (Perciformes: nomeidae) with phylogenetic consideration. Mitochondrial DNA A DNA Mapp Seq Anal. 27(6):4296–4297. 26404517 10.3109/19401736.2015.1082103

